# Aims, methods and preliminary findings of the Physical Activity, Nutrition and Allergies in Children Examined in Athens (PANACEA) epidemiological study

**DOI:** 10.1186/1471-2458-7-140

**Published:** 2007-07-04

**Authors:** Kostas N Priftis, Demosthenes B Panagiotakos, Michael B Anthracopoulos, Anastasios Papadimitriou, Polyxeni Nicolaidou

**Affiliations:** 1 Department of Allergy-Pneumonology, Penteli Children's Hospital, P. Penteli, Greece; 2 Department of Nutrition Science – Dietetics, Harokopio University, Athens, Greece; 3 Respiratory Unit, Department of Pediatrics, School of Medicine, University of Patras, Rio, Greece; 4 Third Department of Pediatrics, Attikon Hospital, School of Medicine, University of Athens, Athens, Greece

## Abstract

**Background:**

To determine the prevalence of asthma symptoms in a sample of Greek children aged 10–12 years, and to evaluate these rates in relation to anthropometric, lifestyle characteristics and dietary habits.

**Methods:**

During 2006, 700 schoolchildren (323 male and 377 female), aged 10–12 years (4^th ^to 6^th ^school grade), were selected from 18 schools located in the greater Athens area. The schools were randomly selected from a list provided by the regional educational offices. To achieve a representative sample the schools enrolled were selected from various region of the Athens area. For each child a questionnaire was completed that was developed for the purposes of the study to retrieve information on: age, sex, school class, other socio-demographic characteristics, anthropometric measurements, dietary habits (through a semi-quantitative Food Frequency Questionnaire) and physical activity status; the presence of asthma and allergies was assessed by the standard ISAAC questionnaire.

**Results:**

The prevalence of wheezing in the past was 25% in boys and 19% in girls, while the prevalence of current wheezing was 9.0% in boys and 5.8% in girls. The prevalence of any asthma symptoms was 27.6% in boys and 20.4% in girls. Multiple logistic regression analysis revealed that increased body weight and sedentary lifestyle is associated with asthma symptoms only in boys.

**Conclusion:**

The present cross-sectional study cannot establish causal relationships between asthma and increased body weight of schoolchildren; however, our findings underline the associations between asthma, increased body weight, and physical activity at population level, and urge for actions that should be taken by public health policy makers in order to prevent these conditions among children.

## Background

Asthma is the leading chronic childhood illness throughout the developed world and places a large burden on affected children and their families. Estimates of current asthma prevalence in the USA indicate that 83 per 1000 (i.e. 6.1 million) children aged 0–17 currently have asthma [1]. In Europe the prevalence of asthma is similar to that observed in the US and has increased decades earlier in Western as compared to Eastern Europe. The International Study of Asthma and Allergies in Childhood (ISAAC) [2] evaluated children 13–14 years-of-age from 56 countries and 6–7 years-of-age from 38 countries. The prevalence of wheezing ranged 4.1–32.1% in the younger age group and 2.1–32.2% in the older age group and was particularly high in English speaking countries and in Latin America. The major differences between populations found in the ISAAC study are most likely due to environmental factors. Many reports underline the importance of monitoring asthma prevalence, health care, and mortality in order to estimate the burden of the disease and to help assess the impact of asthma prevention programs and improvements in health care quality [3-5].

Parallel to the increase of asthma prevalence in children, there is a growing global childhood "obesity epidemic" with a large variation in secular trends across countries [6]. The most significant long-term consequence of childhood obesity is its persistence into adulthood, with all the attendant health risks. The likelihood of such persistence increases with the onset of obesity in late childhood or adolescence and with the severity of obesity [7]. Although the mechanisms responsible for the development of obesity are not fully understood, it has been confirmed that obesity occurs when energy intake exceeds energy expenditure; thus, dietary habits, physical activity, other lifestyle choices and cultural environment as well as a variety of genetic factors significantly influence its development [8].

The association between childhood asthma and body weight has been a matter of particular interest in recent literature and an increasing body of evidence implicates obesity as a major risk factor for asthma, thus linking these two epidemics [9-11]. Indeed, obese children may be prone to receive a diagnosis of asthma as they may complain of shortness of breath on physical exertion sooner than their non-obese peers [12] or they may actually experience increased exercise-induced broncho-constriction as compared to non-obese children [13]. Emerging evidence suggests that asthma symptoms and obesity in children show similar trends in the last few decades [14,15] and that there is greater burden of respiratory symptoms and use of medication among obese children [16-18]. Overweight asthmatic children experience greater limitation of physical activity, while insufficient physical activity increases the risk for higher body mass index, thus perpetuating the vicious cycle of asthma, exercise-induced broncho-constriction, decreased physical activity and increased body weight.

A direct relationship between the prevalence of asthma symptoms, rhinitis and eczema, and dietary factors has been observed [19]; a higher total polyunsaturated fat intake in young sensitized wheezy children has also been reported [20]. There is increasing interest in recent literature with regard to the importance of dietary antioxidant and lipid intake in the expression of asthma during pregnancy and early childhood [21].

In Greece a greater than 4-fold increase in the prevalence of childhood asthma and wheezing has occurred over the last 25 years [22]. A high prevalence of overweight and obesity and a concomitant positive secular change over the last decades has also been observed [23,24], and associated by some investigators with increases in asthma prevalence. Therefore, the Physical Activity, Nutrition and Allergies in Children Examined in Athens (PANACEA) study was designed to determine the prevalence of asthma symptoms among Greek schoolchildren and to evaluate these rates in relation to anthropometric, lifestyle characteristics and dietary habits of the children. This report illustrates the PANACEA study aims, design and methods used, as well as the associations between asthma symptoms and dietary and physical activity characteristics of the participants.

## Methods

The PANACEA study is an epidemiological cross-sectional survey that has three main research hypotheses, i.e. that the prevalence of asthma and allergies in 10–12 year-old boys and girls from Greece is related to their: (a) socio-demographic characteristics, (b) level of physical activity, and (c) dietary habits. Further, it will investigate the interactions between diet, physical activity and socio-demographic characteristics, in relation to the prevalence of asthma and allergic symptoms.

The study has been designed according to the principles of the declaration of Helsinki (1989). The protocol of the study has been approved by the Education Institute of the Hellenic Ministry of Education (approval 29712/G7/2006). All participants were interviewed and evaluated by trained personnel (pediatricians, general practitioners, dieticians and nurses) who used a standard questionnaire with closed-ended questions.

### Study sample

During 2006, 815 schoolchildren aged 10–12 (4^th ^to 6^th ^school grade) years from 18 public schools located in the greater Athens area were recruited to participate in the study. Seven hundred schoolchildren (323 male and 377 female) agreed to enrol into the Study (participation rate 83.5%). The schools were randomly selected from a list of schools provided by the regional education offices. In order to achieve a representative sample the enrolled schools were selected from various regions of Athens.

Participation of subjects was on a voluntary basis; prior to acceptance, children's guardians were fully informed on the objectives and methods of the study and signed an informed consent. Only pre-menstrual girls were included. Children suffering from chronic diseases that prohibited free running, i.e. cyanotic heart disease or severe motor handicaps, were also excluded.

The number of enrolled children was adequate (i.e., statistical power >85%) to evaluate two-sided hypotheses regarding standardized differences between various balanced groups (such as obese *vs*. non-obese children, physically active vs. inactive etc.) greater than 0.5 at the probability level of <0.05.

### Funding

The study was funded by research grants by the Respiratory Unit of the Department of Pediatrics, University of Patras, Greece.

### Socio-demographic and anthropometric measurements

The parents of every child completed a questionnaire that was developed for the purposes of the study and was designed to retrieve information on: age (in years), sex, school class (i.e., 4^th^, 5^th ^or 6^th^), dietary habits,  asthma and other allergic symptoms. The same questionnaire also included information regarding the social status of the family, i.e. educational level of the parents (none, basic education, high school, academic), number of cars within the family, availability of child's own-room and number of siblings as well as information on hospitalizations of the children. A special questionnaire on the children's physical activity was completed at school by children themselves.

Standing height was measured with a Raven Minimeter (Raven Equipment Limited, Essex, United Kingdom) to the nearest 0.1 cm and body weight to the nearest 0.1 kg with a Seca weighing scale (Seca, Hanover, MD) after students had removed their clothes and shoes. Measurements took place at the school setting. Overweight and obesity were defined using the international body mass index cut-off points established for children and youth [25]. These cut-off points are based on health-related adult definitions of overweight (25–29.9 kg/m^2^) and obesity (≥ 30 kg/m^2^), and are adjusted to specific age and sex categories for children. Parents were also asked to report the height and weight of their children.

Children's birth-weight was reported by the parents who were encouraged to use the standard health record booklet that accompanies each child in Greece from birth; the information was entered into the following five categories: <1,500 g, 1500–2,000 g, 2,000–2,500 g, 2,500–3,500 g and >3,500 g. Information on breast-feeding and its duration (in months) was also provided.

Reported height, weight and calculated BMI of both parents were recorded. Parents were classified as normal, overweight (BMI: 25.0–29.9 kg/m^2^) or obese (BMI: ≥ 30.0 kg/m^2^).

### Evaluation of asthma symptoms

In order to evaluate asthma symptoms, the approved Greek version of the International Study for Asthma and Allergies in Childhood (ISAAC) core questionnaire was employed [2,26]. In particular, the evaluation of presence and duration of asthma symptoms was assessed by four questions according to ISAAC classification: 1) if children ever had wheezing, 2) if they ever had disturbed sleep due to wheezing, 3) if they ever had asthma, and 4) if they ever had dry cough at night, except in case of cold or chest infection [2].

### Evaluation of dietary habits

A semi-quantitative Food Frequency Questionnaire (FFQ) that gathers information on a daily and weekly basis was applied to all children. Various foods and beverages commonly consumed in Greece, and habits pertaining to mealtime behaviours were recorded by using 63 detailed descriptive questions. In particular, we measured the weekly or daily intake of dairy products with breakfast, the frequency of breakfast consumption, the frequency of consumption of cereals with breakfast, the daily consumption of meals including snacks, the frequency of consumption of foods outside the home (including school canteens and non home-made meals), the cooking method usually employed by the family, the type of oils/fat consumed, the frequency of snacks consumed and the frequency of consumption of various foods such as: fish, poultry, red meat, eggs, white bread, whole grain bread, potatoes, rice, fruits, vegetables, fruit juices, soda drinks, low-calorie soft drinks, beverages, and of traditional Greek cooked meals. The above mentioned foods and beverages were categorized as follows: (1) "dairy products" that included all kinds of milk, yoghurt and cheese; (2) snacks were categorized into two main types: "salty" snacks (such as hamburger, pizza, hot-dog, toast, cheese pie, spinach pie, all kind of crisps, popcorn) and "sweet" snacks (such as ice-cream, milk shake, all kinds of chocolate, croissant, cakes, biscuits); (3) any type of soda drink; (4) fruit juices (fresh, or ready-made); (5) other beverages (such as tea, chamomile, etc); and (6) traditional Greek cooked foods. Typical serving sizes were employed as units for measurements and for every food/drink item in the questionnaire, a clearly described measure was used (e.g. a can of soft drink, one hamburger, one portion of chicken approximately 150 gr, a bag of crisps, etc). When the FFQ was completed, subjects indicated the average frequency of consumption of the reported amount of each food item. Furthermore, based on the above questionnaire, the daily energy intake of every child was calculated (in kcal). Then all children were categorized into four groups: (a) low (<1,800 kcal), (b) moderate (1,800–2000 kcal), (c) adequate (2,000–2,350 kcal) and (d) high (>2,350 kcal).

### Evaluation of physical activity

Information on the frequency and duration of a variety of physical activities of the children was retrieved and recorded in a validated reliable questionnaire [27]. In particular, questions for this questionnaire were read by the researcher in front of children and they answered questions on how often and at which level of competitive requirement they participate in sports related physical activities such as brisk walking, running, swimming and cycling; non-sports related activities, such as going out with friends, social visits, going to the movies or theatre; and on the amount of time spent on sedentary activities (e.g., watching television, working on a computer, playing video games). The physical activity questionnaire has been previously validated in metabolic equivalence (MET, 1 MET = 3.5 ml/kg/min) by the Department of Sports Science, of the Democritus University of Thrace [27]. According to this questionnaire children were classified as having: very low (<2 MET/min), low (2–3 MET/min), moderate (4–5 MET/min), good (6 MET/min) and very good (>6 MET/min) physical activity.

### Data analysis

Continuous variables are presented as mean ± standard deviation, and categorical variables are presented as absolute and relative frequencies. Contingency tables with the calculation of chi-square test evaluate the associations between the categorical variables. Relationships between genders and continuous variables are tested by the use of Student's t-test (for normally distributed variables), after checking for homoskedacity, or the Mann-Whitney test for skewed variables. The estimates of the relative risks of asthma symptoms are reported by calculating the odds ratios (ORs) and the corresponding 95% confidence intervals (CIs), using multiple logistic regression analysis. The dependent outcome was the presence of asthma symptoms and independent predictors were age, sex, body weight, body mass index, physical activity status, hours of TV viewing or playing video games, and foods consumption. Significant confounders, as well as interactions are retained in the models. Deviance residuals are calculated in order to evaluate the model's goodness-of-fit. All reported probability values (p-values) are based on two-sided tests and compared to a significant level of 5%. STATA 6 software was used for the all calculations (STATA Corp., College Station, Texas, USA).

## Results

Table 1 presents the age-sex distribution of the study sample.

**Table 1:** Distribution of study sample by age group.

Age (years)	Boys	Girls	Total
10	81	59	140
11	117	159	276
12	125	159	284
Total	323	377	700

Table 2 presents various socio-demographic, anthropometric and lifestyle characteristics of the children. Approximately half of the parents reported high academic education (i.e., technological studies or universities). Three out of four children reported that they have their own room, indicating moderate to good financial status. Less than 8% of children had birth weight below 2000 g, and nearly one out of three children was breastfed for more than 3 months. Very low physical activity was reported by 16–17% of boys and girls, while 54% of boys and 46% of girls reported that they participated in moderate to vigorous physical activities during a regular week. The time spent for sports related activities during the week was approximately 4 hours, and a similar amount of time was devoted to non-sports related activities. Moreover, children reported that they spent approximately 2 hours per day watching television or playing video games. Additionally, the frequency of having breakfast was approximately 5 times per week and it is similar between boys and girls, the number of meals are approximately 3 per day, while the number of vegetables and fruits or juices consumed on a weekly basis seems adequate.

**Table 2:** Socio-demographic, anthropometric and lifestyle characteristics of the participants (percentage or mean ± SD).

	Boys (n = 323)	Girls (n = 377)
Parents with academic education	49%	48%
Number of cars per family	1.6 ± 0.6	1.6 ± 0.8
Children with own bedroom	73%	74%
Birth weight < 2500 gr	5.5%	7.5%
Breast fed > 3 months	27%	31%
Body mass index (kg/m^2^)	20.5 ± 3.5	20.2 ± 3.7
Overweight	34%	22%
Obese	9.4%	8.6%
Very low physical activity	15.9%	16.8%
Watching TV or playing video games (hours/day)	2.3 ± 1.3	2.1 ± 1.3
Sports activities (hours/week)	4.1 ± 3.3	3.2 ± 2.7
Non-sports activities (hours/week)	4.0 ± 2.3	4.3 ± 4.3
Frequency of eating breakfast/week	5.2 ± 2.4	5.0 ± 2.6
Number of meals per day	3.2 ± 1.0	3.3 ± 1.4
Eating at school canteen	34%	42%
Eating vegetables per week (portion(s))	17.6 ± 14.7	19.7 ± 15.2
Eating fruits per week (portion(s))	34.4 ± 22.4	38.6 ± 23.4
Daily drinking of fresh juices	43%	40%
Daily drinking of soft drinks	8.6%	6.2%

Overall lifetime prevalence for asthma symptoms was 27.6% in boys and 20.4% in girls (p = 0.027). Table 3 illustrates the prevalence of asthma symptoms in our sample. Twenty five percent of boys and 21% of girls reported chronic rhinitis.

**Table 3:** Frequency (%) of asthma symptoms reported in boys and girls of the study

	Current wheeze	Ever had wheeze	Wheeze disturb sleep	Wheeze limiting speech	Exercise wheeze	Night cough	Ever had asthma	Any asthma symptoms
Boys	29 (9.0%)	75 (25.3%)	3 (3.8%)	4 (4.8%)	14 (4.8%)	44 (14.9%)	43 (13.3%)	89 (27.6%)
Girls	22 (5.8%)	65 (18.5%)	8 (11.6%)	4 (5.48%)	15 (4.3%)	38 (11.0%)	31 (8.2%)	77 (20.4%)
Overall	51 (7.3%)	140 (21.6%)	11 (7.4%)	8 (5.1%)	29 (4.5%)	82 (12.8%)	74 (10.6%)	166 (23.7%)

Multi-adjusted logistic regression analysis revealed that in boys: 5 kg increase of body weight increased the odds of asthma symptoms by 13% [95% CI 1.02, 1.25], one hour of increase of TV viewing or playing videogames increased the odds of asthma by 10% [95% CI 1.01, 1.04]. Moreover, boys with asthma symptoms are 2.2-times more likely [95% CI 1.34, 3.54] to not participate in any physical activity than boys without any asthma-related symptoms. Body weight and TV viewing or playing with videogames were not associated with asthma symptoms in girls. Finally, girls that did not participate in any activity during a week were 63% more likely to have asthma symptoms [95% CI 0.86, 3.11] than girls who participated in physical activities. No significant associations were observed between specific food consumption or energy intake and prevalence of asthma symptoms.

## Discussion

In this paper we present the aims, design and preliminary results of an epidemiological study on asthma, increased body weight, and physical activity among schoolchildren that has been conducted in the Athens greater area, Greece (i.e., the PANACEA study). We also present the basic characteristics of children participating in the study regarding socio-demographic status, dietary and other lifestyle habits and the reporting of asthma symptoms. To the best of our knowledge this is the first epidemiological study that evaluates dietary habits and physical activity in relation to the prevalence of asthma symptoms among school-aged children in Greece.

There have been many lifestyle changes of Greek children in the last few decades that may have contributed to the increase in the prevalence of childhood asthma. It has been reported that asthma hospital admissions of children in Athens rose by 271% from 1978 to 2000 [28]. These trends are supported by another recent publication regarding the prevalence of asthma in another Greek region (i.e. the city of Patras), where prevalence of current asthma has risen among schoolchildren from 1.5% to 6.9% over a period of 25 years, from 1978 to 2003 [22]. All these studies are in accordance with reports from the ISAAC steering committee regarding the emerging epidemic of childhood asthma throughout the world [29]; however, prevalence of asthma in Greece is ranking among the lowest worldwide [29]. This outstanding observation may be related to factors particular to this region, such as diet.

The prevalence of obesity has reached alarming levels worldwide, especially among children. Moreover, international surveys have highlighted the fact that overweight and obesity among Greek adolescents are currently a major health problem. In particular, the findings from a survey conducted in 34 countries in 2001–2002 showed that in Greece the prevalence of overweight and obesity in adolescents was 15% and 2%, respectively [30]. Likewise, Lissau et al., within the framework of the Health Behaviour in School-aged Children Obesity Working Group, reported that the highest prevalence of overweight in adolescents was observed in the United States, Ireland, Greece and Portugal, among 13 countries included in the study [31]. Our findings support these previous reports since approximately 4 out of 10 schoolchildren in our survey were found to be overweigh or obese. Future analyses of the data accumulated by the PANACEA study may help to sort out some of the possible risk factors in modern living that result in obese children.

The FFQ has been found reliable in several analyses performed by our group, but has not been validated for nutrient intake. Usual dietary intake of the participants over the week preceding enrolment was assessed by using 63 detailed descriptive questions, which included various foods and beverages that are commonly consumed in Greece. Lack of significant associations between food groups intake and asthma prevalence need further attention, since several dietary factors interact and may influence outcome with an unpredictable way. The methods used to evaluate physical activity in children and adolescents are being widely discussed, since the accurate measurement is critical for determining current levels of physical activity [32]. A number of different measurement approaches have been described for assessing children's activity, but no specific method can be identified as the best option for all studies. The selection of an appropriate instrument depends on the specific research question being addressed as well as the relative importance of accuracy *versus *practicality [33,34]. We used a self-report method, as it is considered more appropriate than objective instruments for a large epidemiological study, such as ours. The physical activity and lifestyle questionnaire employed has been validated and found to be reliable in Greek schoolchildren older than 10 years [27].

### Limitations

The PANACEA study is a cross-sectional one and therefore has the inherent limitation of not being able to make causal relationships; the value of its findings lies in stating hypotheses. Moreover, a major issue in our analyses is that of reverse causality as respiratory symptoms may themselves alter activity, weight and diet. Although rather small, as compared to other studies, our sample size should be considered adequate for evaluating associations. However, it is not large enough in order to accurately reflect the prevalence of health related conditions (i.e. asthma, increased body weight and level of physical activity) in the investigated population. The high frequency of children from high socioeconomic background recruited in the study is most likely the result of participation bias; therefore, the importance of risk factors detected is limited to this population group. In addition, certain information, such as on breastfeeding, were retrieved from parents' reports, and therefore are subjects to recall bias.

## Conclusion

Our findings underline the importance of asthma and increased body weight at a population level, in Greece, and urge for actions that should be taken from public health policy makers in order to prevent this condition among children. We anticipate that further analysis of the available data will provide current, novel and valuable information on the relations of dietary, lifestyle, physical activity and clinical characteristics with asthma, other allergies and obesity in Greek schoolchildren. Possible dietary and physical activity particularities -e.g. Mediterranean diet, specific sports activities- may offer important clues on the role of certain foods or exercise in the development of asthma symptoms.

## Competing interests

The author(s) declare that they have no competing interests.

## Authors' contributions

KNP is the principal investigator, had the concept, designed and co-authored the study; DBP is co-principal investigator, co-designed, and is the principal author of the paper; MBA participated in the design and co-authored the paper; AP and PN participated in the design of the study.

## Pre-publication history

The pre-publication history for this paper can be accessed here:


